# Cardiovascular tissue banking in Croatia

**DOI:** 10.1186/1749-8090-8-S1-P86

**Published:** 2013-09-11

**Authors:** I Safradin, B Golubic Cepulic, M Golemovic, R Habekovic, M Borojevic, M Skific, D Jelasic, I Marekovic, S Ivankovic, B Biocina

**Affiliations:** 1 Department of Cardiac Surgery, University Hospital Centre Zagreb, Zagreb, Croatia; 2 Department of Transfusion Medicine and Transplantation Biology, University Hospital Centre, Zagreb, Croatia; 3 Department of Pathology and Cytology, University Hospital Centre Zagreb, Zagreb, Croatia; 4 Department of Microbiology, University Hospital Centre Zagreb, Zagreb, Croatia

## Background

The Croatian Cardiovascular Tissue Bank (CTB) was established in June 2011 at University Hospital Centre Zagreb (UHC). Cardiovascular tissues processed in CTB were retrieved and processed according to validated protocols and in compliance with laws and regulations applied in tissue establishments.

## Methods

Cardiovascular tissue allografts (CVA) were retrieved from recipients of heart transplants (RHT) and multiorgan donors (MOD) which fulfill donor selection criteria. Processing of CVA was performed in CTB clean room (GMP Class B). Initial processing included surgical dissection, measurements, morphology and functional assessment and collection of quality control samples. CVA were incubated in antibiotic containing medium (vancomycin, lincomycin, polymyxin B sulfate) for 24-48 hours. Then CVA were cryopreserved in solution containing 10% of dimethylsulphoxyde and stored in vapor phase of liquid nitrogen. Cryopreserved tissues remained in the quarantine until all quality control results were collected.

## Results

During time period from June 2011 to March 2013, 63 cardiovascular tissue allografts were retrieved from 35 donors (17 RHT and 18 MOD). 32 CVA were accepted for clinical use. Accepted tissues were as follows 15 aortic valves, 13 pulmonary valves, 4 thoracic aortas. 10 CVA remained in quarantine and 21 CVA were discarded. Reasons for CVA to be discarded were microbiological, serological and morphological. 12 CVA (9 aortic valves and 3 thoracic aortas) were successfully transplanted since June 2012. Indications were infectious (7 patients), non-infectious (2 patients) and congenital (1 patient).

## Conclusion

The cardiovascular tissue bank was successfully established at UHC Zagreb. CTB needs to work on the rising of public and surgeons awareness about cardiovascular tissue donation in order to achieve large selection of high quality cardiovascular tissue allografts. Initial clinical results are encouraging.

